# Characterizing the transplanar and in-plane water transport properties of fabrics under different sweat rate: Forced Flow Water Transport Tester

**DOI:** 10.1038/srep17012

**Published:** 2015-11-23

**Authors:** K. P. M. Tang, K. H. Chau, C. W. Kan, J. T. Fan

**Affiliations:** 1 Institute of Textiles and Clothing, the Hong Kong Polytechnic University, Hung Hom, Hong Kong; 2 Department of Fiber Science and Apparel Design, College of Human Ecology, Cornell University, Ithaca, 14853, NY, United States

## Abstract

The water absorption and transport properties of fabrics are critical to wear comfort, especially for sportswear and protective clothing. A new testing apparatus, namely Forced Flow Water Transport Tester (FFWTT), was developed for characterizing the transplanar and in-plane wicking properties of fabrics based on gravimetric and image analysis technique. The uniqueness of this instrument is that the rate of water supply is adjustable to simulate varying sweat rates with reference to the specific end-use conditions ranging from sitting, walking, running to other strenuous activities. This instrument is versatile in terms of the types of fabrics that can be tested. Twenty four types of fabrics with varying constructions and surface finishes were tested. The results showed that FFWTT was highly sensitive and reproducible in differentiating these fabrics and it suggests that water absorption and transport properties of fabrics are sweat rate-dependent. Additionally, two graphic methods were proposed to map the direction of liquid transport and its relation to skin wetness, which provides easy and direct comparison among different fabrics. Correlation analysis showed that FFWTT results have strong correlation with subjective wetness sensation, implying validity and usefulness of the instrument.

Water absorption and transport properties of fabrics are important in determining the thermophysiological comfort of apparel and health-care products. Textile fabrics, located in-between skin and the ambient environment, determine the efficiency of sweat leaving our body. If the resistance to water transport of a fabric is too high, sweat cannot be wicked away from the skin efficiently, creating dampness and clamminess discomfort. Problems will get even worse under high activity level or in hot environment since heat loss depends on absorption property of fabric and also the evaporation of sweat^1^. Therefore, the evaluation of water absorption and transport ability of textiles is important for the optimization of sportswear, functional clothing or other health-care products. Properties such as wicking across (in-plane wicking) and through the plane of the material (transplanar wicking) should be investigated. In-plane wicking controls the spreading area in a fabric which facilitates the evaporation of sweat within the fabric. Transplanar wicking transports the sweat away from the skin so as to minimise the wetness sensation. A measurement technique which is capable of characterising these two directions of wicking is desirable.

A number of water absorption and transport tests have been developed^2^,^3^,^4^,^5^,^6^,^7^,^8^,^9^,^10^ (principles of the test can be found in Supplementary Table S1 online and Tang et al’s review article^11^). Some can provide only general characterization of surface hydrophobicity/hydrophilicity, others require delicate equipment (e.g. spectrophotometer^12^, tomography device^13^,^14^, Nuclear Magnetic Resonance spectrometer^15^, MRI scanner^16^,^17^). Despite this, existing testing methods do not simulate the end-use conditions of fabric (e.g. do not wet the fabric continuously^18^ or do not deliver water to fabric in proper direction^19^,^20^,^21^), could not differentiate the direction of water spread^18^,^22^,^23^ and could only apply to certain types of fabrics (e.g. fabrics in plain colour only^24^,^25^). The testing processes in some cases are complicated^5^,^12^,^17^,^26^,^27^ and most of them are incapable of obtaining sufficient information on the water transport property (e.g. only in-plane wicking property was investigated but not transplanar wicking)^6^,^18^,^28^. Take Gravimetric Absorption Testing System (GATS) as an example^29^, the amount of water absorption in a fabric layer was measured against time. However, it does not give any information about the transplanar water flow. Miller and Friedman^30^ mentioned that in-plane wicking rate may not be reliable for predicting flow in transplanar direction, hence a measurement method which can measure two directions of wicking is desired. Additionally, for some existing methods^23^,^31^,^32^, water was applied to the fabric based on its water absorption capacity (i.e. demand wetting principle). The recently developed instrument, Spontaneous Uptake Water Transport Tester (SUWTT)^33^, is also based on this principle, so more water is supplied to the more water absorbent fabrics during the tests, which tends to differ from the actual end-use conditions. In other existing tests^18^,^21^, although fixed quantity of water was applied, the rate of water supply could not be quantified. In fact, the degree of comfort of fabric might vary with the end use condition. Fabrics with low water absorption capacity but moderate water transport property may be favourable at relatively low sweating levels. It is, however, less desirable under high sweating levels. Sweat may accumulate on skin surface or even drop off from skin which may affect body heat dissipation. Hence, extra care is required when selecting fabric for particular use. In general, thermophysiological comfort is a result of combined effects of the wearer (e.g. metabolic rate and emotion), his/her clothing and the external environment (e.g. air temperature, air velocity, humidity and radiant temperature)^34^. Once the harmony among these three components is upset, a sensation of discomfort will be evoked. In this study, environmental condition was set to be constant (20 + 1 °C and 65 + 5% R.H.) while the effect of clothing material was investigated under different sweat rates. Supplementary Table S2 online gives a summary about the sweat rate under different activity and environmental condition. With reference to Supplementary Table S2 online and various standards^35^,^36^, Supplementary Table S3 online gives an estimate of sweat rate under different level of work. Reader can refer to these tables when choosing fabrics for particular end-use.

The instrument developed, FFWTT, was based on gravimetric and image analysis technique to measure the quantity of water flow in both transplanar and in-plane directions of a fabric. The weight of water uptake, the spreading area and the water content of the sample were examined. In this paper, the principle, testing procedures and measurement parameters of the FFWTT are introduced. Additionally, the usage of FFWTT was demonstrated by investigating the water absorption and transport performance of the 24 types of fabrics with different fabric construction, yarn type, fibre content and varying concentration of water repellent finish. Details of each group and the specifications of each fabric are summarized in Supplementary Table S4 online. For easy and direct comparison between a number of fabrics, two graphic methods were proposed to map the direction of liquid transport and its relation to skin wetness. The effect of water flow rate on water absorption and transport property of fabrics was also investigated. Last but not least, the accuracy, validity and reproducibility of the FFWTT were examined and discussion about these aspects can be found in supplementary information online.

## Methods

### Design and configuration of the experimental set up

**Figure 1:**
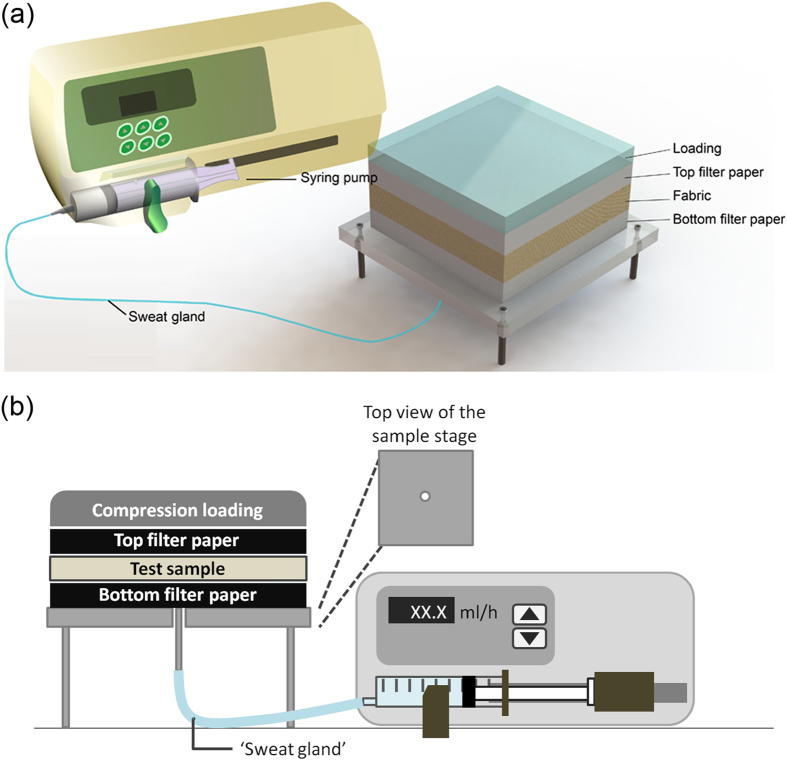
Experimental set up of FFWTT. The test sample was placed in-between two filter papers and a compression loading was put on top of them. Syringe pump was utilised to supply water to the sample at a constant flow rate. The sample stage and the syringe pump were connected by the water tube. (**a**) 3D drawing of FFWTT, and (**b**) Schematic diagram of FFWTT.

FFWTT, diagrammed in Fig. 1, can be divided into two parts: (i) sample stage (square: 12 cm × 12 cm) and (ii) water supply part. The sample stage was made by polytetrafluorethylene (PTFE) to minimize interfacial wicking between the sample and the stage. On top of the stage, the testing specimen was placed in-between two layers of standard material (ADVANTEC^®^ filter paper No.1) for examining the direction of water spread. The use of this particular filter paper could be attributed to the following reasons: (i) very good water absorption and transportation, (ii) guaranteed wetting performance with high reproducibility, (iii) flat surface with random fibre arrangement, and (iv) easily purchased at relatively low cost. A compression loading was placed above the sample to ensure even and reproducible contact between the layers. For the water supply part, syringe pump was utilized which enables constant rate of water supply and the rate of water supply is adjustable. The syringe pump and the sample stage were connected by a silicone tube with outer diameter of 3 mm and inner diameter of 1 mm. This tube, acted as a sweat gland, was used to deliver ‘perspiration’ away from the body. To minimize the gravitational effect on absorption, water was supplied from the bottom to the back side of the sample.

### Principle of the test

Throughout the experiment, water supply was continuous under constant water flow. By switching the setting in the syringe pump, the water flow rate is adjustable to simulate different sweating levels based on the specific end-use. The physiological response (sweat rate) is assumed constant. This is useful when selecting fabric for a particular activity and environmental condition. In this study, water flow rate of 3 ml/h, 10 ml/h and 40 ml/h was investigated which simulates different levels of sweating. The relationship between water flow rate and sweat rate is demonstrated by equation (1). This assumes that sweat was uniformly distributed in our body whilst water was uniformly distributed in the entire fabric surface. Given that the surface area of the testing sample is 0.0144 m^2^ and the surface area of human body is 1.8 m^2^ 37, the corresponding sweat rate of human body is 375 ml/h (208 ml/m^2^/h) when the injection speed of syringe pump is 3 ml/h. For 10 ml/h and 40 ml/h water supply, the corresponding sweat rate is 1250 ml/h (694 ml/m^2^/h) and 5000 ml/h (2778 ml/m^2^/h), respectively. With reference to Supplementary Table S3 online, the injection speed of syringe pump of 3 ml/h represents gentle walking or driving. On the other hand, the injection speed of 10 ml/h (i.e. sweat rate of 1250 ml/h) represents high speed running. For the injection speed of 40 ml/h, it was set with reference to the water supply rate of another tester, Spontaneous Uptake Water Transport Tester (SUWTT), of which the rate of water supply is fabric-dependent and ranged from 37.37 to 80.66 g/h as shown in Supplementary Figure S5 online. This simulates extremely high level of work and approximates to the highest sweat rate of human.


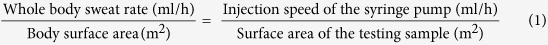


The targeted injection amount of water was set as 0.6 ml and the injection time varies with the injection speed as calculated by equation (2). The higher the water flow rate, the shorter the injection time is.


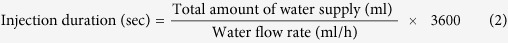


Based on equation (2), the duration of water supply is 720 seconds at 3 ml/h water supply, 216 seconds at 10 ml/h water supply and 54 seconds at 40 ml/h water supply.

Once sweating occurs, the injected water was first contacted with the bottom filter paper, followed by the fabric and the top filter paper. Here, the bottom filter paper acted as a simulated skin helps to dispense the injected water from the sweat gland (water pipe) while the combination with the top filter paper was used for characterizing the transplanar water transport behaviour. Since water absorption and transport ability of the filter papers and the test sample are different, the water gain in each layer varies, contributing to between-layer difference and between-fabric difference. The less or slower absorptive the fabric, the more the water remained on skin and the poorer the wear comfort it provides.

Apart from the water flow rate, the pressure loading applied onto the sample was also adjustable in this set up. The applied pressure might be set at a higher level for tight-fitted garment and at a lower level for loose-fitted garment. Preliminary experiments suggest that the applied pressure should not be too low (around 0.5 g/cm^2^) to ensure reproducibility of the test. The selection of pressure loading is demonstrated in the supplementary information online. With reference to reproducibility and testing sensitivity, external loading of 360 g (2.5 g/cm^2^), which is the same as the setting for the standardized water absorption tester - Gravimetric Absorption Testing System (GATS)^29^, was chosen. This loading simulates the condition when a tight-fitted garment is worn.

### Testing procedures

Before the test, the sample was put onto the sample stage and compressed with an external loading. The syringe pump was switched on for a predetermined duration. After injection, the compression plate was removed and layers of textiles were separated immediately. The water gain in each layer was measured by an electronic balance. The spreading pattern of each layer was scanned by an optical scanner and the wetting area in different layers was calculated.

### Measurement parameters

In this study, the targeted amount of water injection (i.e. 0.6 ml) was determined with reference to the relevant test in the literature^24^. Since termination of the test was controlled manually, there might be little variation in the injection amount. In order to minimize this variation, the amount of water absorbed by each layer was presented as fraction value. **Fraction of water absorbed by a specific layer** was calculated by the water absorption mass in a specific layer divided by the total absorption amount of the three layers, as shown in equation (3).


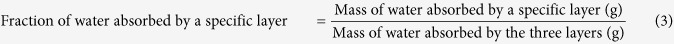


The transplanar water flow was determined by the water distribution in the top and bottom filter paper. By dividing the amount of water absorption in the top filter paper with the bottom one, an index called ‘**Transplanar ratio**’, which has been developed in our previous study^33^, can be determined using equation (4). It is anticipated that this ratio could tell the transplanar wicking ability of the fabric. The higher the ratio, the more the water wicked away from the skin surface and the less the clammy sensation it provides.


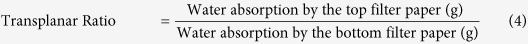


In addition to the gravimetric measurement, the **wetted area** of each layer, which reflects the in-plane wicking property of fabric, was additionally measured and expressed in cm^2^. The larger the spreading area, the greater the area for sweat evaporation.

**Water content** measures the amount of water within the sample. The calculation of water content of fabric is defined in our previous work^33^ and its calculation is shown in equation (5). This parameter is affected by the geometry of the sample with its wetted area, thickness and porosity being considered. In study conducted by Ghali *et al.*^38^, water content is defined as the fraction of the void space that is filled with liquid. At 100% water content, all void space in the sample is filled with liquid. At 0% water content, there is no liquid presenting in the sample excluding the moisture regain from air. In general, the higher the water content within a fabric, the less comfortable it will be.


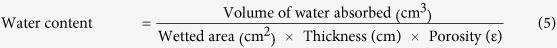


## Results and Discussion

### Investigating the usage and sensitivity of FFWTT

**Figure 2:**
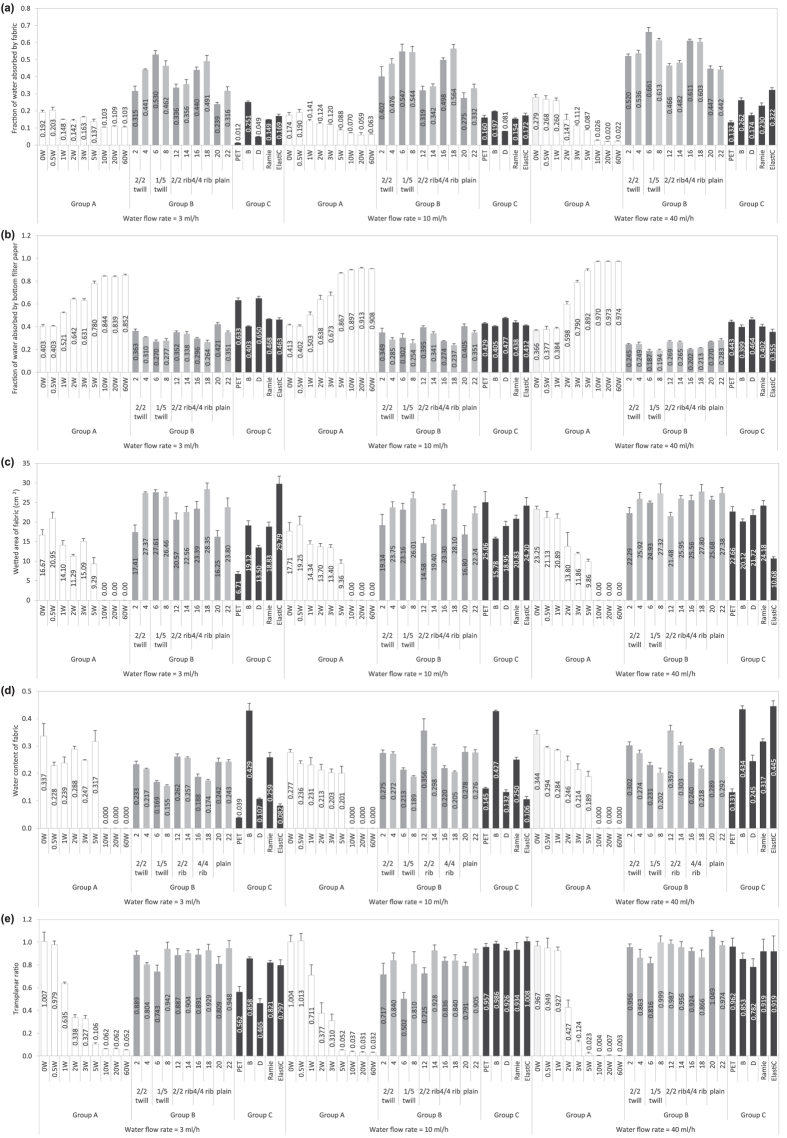
FFWTT results of various fabrics. The bars in white colour indicate the result for group A’s samples while the bars in grey and black denotes group B’s and group C’s samples, respectively. The error bars represent mean ± S.D. of five samples. (**a**) Fraction of water absorbed by fabric. (**b**) Fraction of water absorbed by bottom filter paper. (**c**) Wetted area of fabric. (**d**) Water content of fabric. (**e**) Transplanar ratio.

The measurement results of FFWTT test are illustrated from Fig. 2 (a–e). The significance level of the statistical analysis conducted in this study was set at 0.05. The discussion of this section is divided according to the grouping of the fabrics. Group A fabrics, with varying surface hydrophobicity, aimed at investigating how well FFWTT distinguishes fabrics having different levels of water repellent finishes. Group B aimed at investigating the sensitivity of the instrument in differentiating fabrics with different fabric structures and yarn types. The microscopic images of these fabrics and its yarn arrangement are shown in Supplementary Figure S2 online. The incorporation of Group C’s fabrics, made of different materials, aimed at increasing the variability of the dataset.

### Group A – Fabrics with controlled hydrophobicity

**Figure 3:**
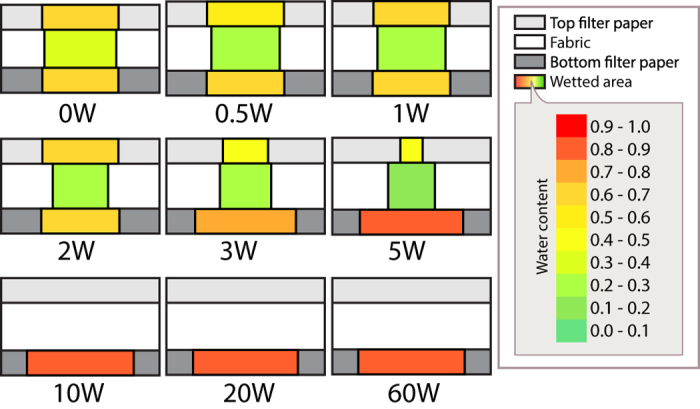
Regional mapping of water distribution within the three layers. The water distribution within the three layers for Group A’s samples, as a result of 40 ml/h water supply, is exemplified in this graph. The box in pale grey colour denotes the top filter paper while the box in white is the fabric layer and the box in dark grey is the bottom filter paper. The thickness of each layer is in proportion with its actual thickness. Additionally, the wetted area of each layer is in proportion with its actual spreading area. The spread of water in each layer was highlighted with different colours corresponding to its water content. Layer with very high water content is presented in red. Layer with moderate water content is presented in yellowish colour whilst layer with very low water content are marked in green.

Fabrics in this group are treated with different concentration of water repellent finishing agent. With increasing concentration of water repellent finish, its fraction of water absorbed by fabric layer (as shown in Fig. 2(a)), wetted area of fabric (as shown in Fig. 2(c)) and transplanar ratio (as shown in Fig. 2(e)) decreases gradually. Figure 3 exemplifies a way for portraying the distribution of water within the three layers and the water content of each layer based on the FFWTT results. It shows that water repellent agent is unfavourable for both in-plane and transplanar wicking. On the other hand, fraction of water absorbed by the bottom filter paper increases with concentration of water repellent finishing (as shown in Fig. 2(b)). This suggests that more water may be left on skin when the hydrophobicity of the fabric is high. For fabric ‘10W’, ‘20W’ and ’60W’, the wetted pattern could not be observed and so their wetted area is zero and water content is undefined.

### Group B – Fabrics with different fabric structure and yarn type

From Fig. 2(a,e), it can be observed that the water absorption and transport property of Group B’s fabrics varies with fabric structure and yarn type. Between-groups analysis of variance (ANOVA) test was performed to test for the ‘main effect’ for each independent variable (i.e. ‘fabric structure’ and ‘yarn type’) on the dependent variable. The ANOVA results, as shown in Supplementary Table S7 online, suggest that the effect of fabric structure is significant in all measured parameters for the three water flow rate studied (p < 0.05) except for the transplanar ratio measured at 3 ml/h water flow (p = 0.056>0.05). The effect of yarn, on the other hand, is significant in all measured parameters for 3 ml/h and 10 ml/h water supply (p < 0.05) but only in wetted area and water content of fabric for 40 ml/h water supply (p < 0.05). It suggests that FFWTT is a sensitive measurement method.

Post hoc tests using Scheffé's method were performed and the results are summarised in Supplementary Table S8 online. The following discussion suggests that the finding from FFWTT is rational. For the plain fabrics (i.e. fabric ‘20’ and ‘22’), due to its lower thickness (shown in Supplementary Table S4 online), its water absorption capacity is lower than the other fabric structures and so does the fraction of water absorbed in the fabric layer (as shown in Fig. 2(a)). Because of its relatively low water absorption capacity, much water may stay in its bottom filter paper and hence fraction of water absorbed in its bottom filter paper is higher (as shown in Fig. 2(b)).

On the other hand, post hoc tests, shown in Supplementary Table S8 online, indicate that fraction of water absorbed by the fabric layer in the 1/5 twill fabrics (i.e. fabric ‘6’ and ‘8’) is higher than the rest. This could attribute to higher fabric porosity and thickness of the 1/5 twill fabric (as mentioned in Supplementary Table S4 online) which is likely to accommodate much water. In addition, the water content for fabrics with 1/5 twill structure is lower than the other structures (as shown in Fig. 2(d)). This might attribute to the less intersection points in this structure (as shown in Supplementary Figure S2 online) which facilitates wicking within the fabric.

As for the wetted area of fabric, 4/4 rib fabrics (i.e. fabric ‘16’ and ‘18’) got the largest wetted area in general as shown in Supplementary Table S8 online. For this structure, four weft yarns were grouped together intermittently, forming straight capillaries in weft direction (illustrated in Supplementary Figure S2) which are likely to promote liquid flow and this may explain the largest wetted area observed.

Figure 2(c) also suggests that the wetted area for the fabrics made by 45/2 s cotton yarn (i.e. Fabric ‘4’, ‘14’, ‘18’, ‘22’) is generally larger than the one made by 20/1 s cotton yarn (i.e. Fabric ‘2’, ‘12’, ‘16’, ‘20’). Greater number of smaller inter-yarn pores existed in fabrics woven by 45/2 s cotton yarn (i.e. two thinner yarns twisted together to form a weft yarn) and capillary theory suggests that smaller pores result in higher capillary pressure and enhance liquid spreading distance^39^. This explains why larger wetted area is observed for fabrics made by 45/2 s cotton yarn. As a result, the water content of 45/2 s fabric is also lower than the 20/1 s cotton one as shown in Fig. 2(d).

### Group C–Fabrics with different geometrical properties and fibre content

The five fabrics in this group are woven by different materials and yarns. Fabric ‘PET’ is made by polyester. It is commonly known that the synthetic bulk of polyester does not absorb water whereas cotton, hygroscopic material, absorbs water readily^40^,^41^. Water may wick along the inter-fibre spaces in polyester yarn instead of absorbing by the fibre, so fraction of water absorbed by the non-hygroscopic polyester fabric (‘PET’) is generally lower than the hygroscopic cotton fabrics.

### Mapping liquid spreading and skin wetness

**Figure 4:**
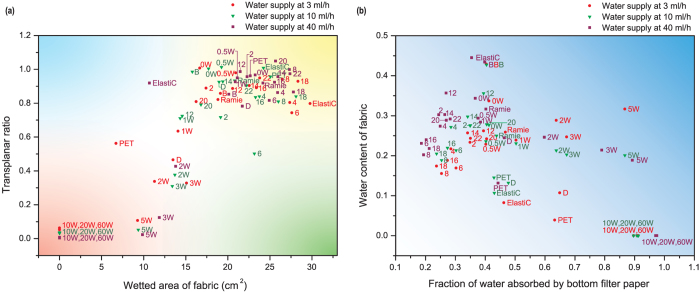
Mapping the direction of liquid transport and skin wetness. The results measured at 3 ml/h water supply are presented in red circles while green triangles and purple squares denote the result measured at 10 ml/h and 40 ml/h water flow, respectively. (**a**) Mapping the direction of liquid transport. Fabrics which are close to the green zone have good in-plane wicking property but poor transplanar wicking. The points which are close to the blue zone have good transplanar wicking property but poor in-plane wicking performance. The ideal fabrics, which have excellent in-plane and transplanar wicking properties, are located in the yellow zone. The most discomfort fabrics are situated close to the origin of coordinate (i.e. red zone). (**b**) Mapping skin wetness. This plot is filled with colour with gradient change from white to blue. The points which are close to the origin of coordinate (i.e. white zone) give a dry skin surface. Inversely, the points which are far away from it give a wet skin surface.

This section provides two mappings for describing the water transport properties of fabrics in response to the direction of spreading and skin wetness. These can be used for ease of comparison between a large group of fabrics. In general, a comfortable fabric should have excellent transplanar wicking (i.e. transplanar ratio is high) and in-plane wicking property (i.e. spreading area of fabric is large) which could facilitate faster evaporation and leave a dry skin surface. Figure 4(a) maps these two important determinants (i.e. the direction of liquid spreading) in a 2-D diagram. For example, fabric ‘18’ situated in the yellow zone is comfortable in terms of both in-plane and transplanar wicking. On the other hand, fabric ‘10W’, ‘20W’ and ‘60W’ are the most discomfort one which has poor transplanar and in-plane wicking property. Figure 4(b) plots water content of fabric against fraction of water absorbed by bottom filter paper and is related to skin wetness. Larger value in both factors contributes to wet skin surface. For fabric ‘5W’, higher proportion of water was absorbed by the bottom filter paper while its water content is also high, suggesting a wet skin surface. For fabric ‘10W’, ‘20W’ and ‘60W’, although its water content is low (water cannot penetrate into the fabric due to its hydrophobicity), majority of water stayed in the bottom filter paper and so giving a wet skin surface. For fabric ‘8’ and ‘18’ (situated close to the white zone), its water content and fraction of water absorbed by the bottom filter paper is low and so it gives a dry skin surface.

### Water absorption and transport properties of fabrics – ‘Sweat rate’ dependent

**Figure 5:**
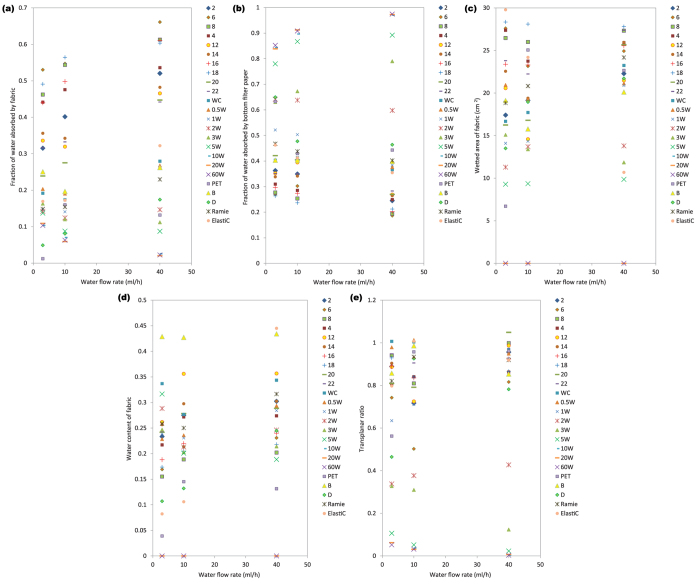
Results of various water absorption and transport properties under different water flow rate. (**a**) Fraction of water absorbed by fabric. (**b**) Fraction of water absorbed by bottom filter paper. (**c**) Wetted area of fabric. (**d**) Water content of fabric. (**e**) Transplanar ratio.

Figure 5 shows the water absorption and transport properties under different sweat rate and the results suggest that it is ‘sweat rate’-dependent. For example, some fabrics might have good wicking property under lower flow rate, but poor performance under faster water flow. These fabrics might be good for casual wear, but not for active sportswear. Take fabric ‘ElastiC’ as an example, this blended fabric (mixed with cotton, spandex and polyester) have excellent in-plane wicking at lower water flow rate. Its wetted area is 29.79 cm^2^ at 3 ml/h (i.e. the 1^st^ among the 24 fabrics) and 24.20 cm^2^ at 10 ml/h (i.e. the 4^th^ among the 24 fabrics). However, further increase the water flow rate will reduce the wetted area of fabric sharply and its wetted area is just 10.68 cm^2^ at 40 ml/h (i.e. the 20^th^ among the 24 fabrics).

Inversely, some non-hygroscopic fabrics might be able to transport water abundantly under faster water flow. However, water may not wick across or through these fabrics easily under lower water flow rate. Take fabric ‘PET’ as an example, its transplanar wicking property is comparatively poor under 3 ml/h water flow (i.e. the 17^th^ among the 24 fabrics) and its transplanar ratio increases sharply to 0.962 at 40 ml/h water flow (i.e. the 6^th^ among the 24 fabrics). Similarly, its wetted area is as low as 6.71 cm^2^ at 3 ml/h (i.e. the 21^st^ among the 24 fabrics) and increases dramatically to 22.66 cm^2^ at 40 ml/h (i.e. the 11^st^ among the 24 fabrics).

The above cases suggest that water absorption and transport property of fabrics varies with ‘sweat rate’ and it confirms the necessity to test under different ‘sweat rate’ with reference to the specific end-use.

## Summary

A new instrument was introduced to measure the water transport of fabrics in both the transplanar and in-plane direction. The test sample was placed in between two filter papers and a syringe pump with adjustable, continuous and constant rate of water supply was utilized to simulate different sweat rates. The water transport properties were determined by monitoring the absorption amount as well as the spreading area in each layer. When majority of water was transported to the top filter paper, it demonstrates excellent transplanar wicking property of the fabric whereas a large spreading area of fabric implies excellent in-plane wicking property. On the other hand, if water was concentrated in the bottom filter paper (simulated skin layer), it implies moist fabric-skin interface and poor wear comfort. Experimental results show that FFWTT has high sensitivity, accuracy and reproducibility while validity of FFWTT has also been confirmed by investigating its relationship with subjective wetness sensation (discussion about accuracy, reproducibility and validity of FFWTT can be found in Supplementary information online).

Regional mapping of liquid spreading within the three layers was examined. It can help to visualise the distribution of water and the water content of each layer. Detailed information could be obtained at a glance. Additionally, two mapping methods were proposed for graphically representing the water transport properties in both directions and its relation to skin wetness. These can be used for ease of comparison between a large group of fabrics.

The uniqueness and advantages of FFWTT include:

- Capability of simulating the wearing condition under different sweat rate,
- Capability of simulating the wearing condition with differential stress levels (for instance, the pressure applied to sock fabric might probably be higher than that for a loose t-shirt),
- Capability of tracing the direction of water transport with the ability to estimate the amount of water left on skin when sweating,
- Versatility in terms of the types of fabrics can be tested (including the hydrophobic fabrics, towels, diapers, moisture management fabrics or fabrics having very high water absorption rate),
- Efficiency in testing (The test takes only short time), and
- Simple instrumental setup.

Detailed comparisons of FFWTT against the recently developed SUWTT is summarised in Supplementary Table S11 online. Compared with existing testing methods, FFWTT has improved in many aspects as shown in Supplementary Table S12 online.

## Additional Information

**How to cite this article**: Tang, K. P. M. *et al.* Characterizing the transplanar and in-plane water transport properties of fabrics under different sweat rate: Forced Flow Water Transport Tester. *Sci. Rep.* **5**, 17012; doi: 10.1038/srep17012 (2015).

## Supplementary Material

Supplementary Information 1
