# Supplementary material for: Characterizing the transplanar and in-plane water transport properties of fabrics under different sweat rate: Forced Flow Water Transport Tester
Source: Sci Rep. 2015 Nov 23;5:17012. doi: 10.1038/srep17012 (PMC4655317; doi:10.1038/srep17012)
Supplement: Supplementary Information 1 [file srep17012-s1.pdf]

Supplementary Information For:

Characterizing the transplanar and in-plane water transport property of fabrics under different sweat rate: Forced Flow Water Transport Tester

K. P. M. Tang[1], K. H. Chau[1], C. W. Kan[1*] and J. T. Fan[1,2**]

[1] Institute of Textiles and Clothing, the Hong Kong Polytechnic University, Hung Hom, Hong Kong

[2] Department of Fiber Science and Apparel Design, College of Human Ecology, Cornell University, Ithaca, 14853, NY, United States

*Corresponding author: tccwk@polyu.edu.hk

**Corresponding author: jf456@cornell.edu

Introduction

Supplementary Table S1. Various conventional and newly developed water absorption and transport test methods

Test methods	Testing principles	Measurement parameters
Spontaneous Uptake Water Transport Tester (SUWTT) [1]	Water was supplied to the sample continuously which simulates profuse sweating. The mass of water supply depends on the absorbency of the sample. In 1-layer set up, fabric was placed onto the sample podium and its water absorption rate, spreading area and water content was recorded. In 3 layer set up, the test fabric was placed in-between two filter papers and the distribution of water in each layer was measured. Gravimetric and image analysis technique were adopted for the measurement.	1-layer set up<br>- Water absorption rate (g/s)<br>- Spreading area of fabric (cm^2)<br>- Water content of fabric<br>3-layer set up<br>- Transplanar ratio<br>- Mass of water absorbed by bottom filter paper (g)
Wettability test (AATCC 79) [2]	A drop of water was delivered from a fixed height onto the test sample. The time it takes for the drop of water to disappear was taken as a measure of the wettability of fabric and it was recorded by visual observation. The shorter the time, the much wettable the fabric is.	- Water absorption time (s)
Vertical wicking test (AATCC 197) [3]	A preconditioned strip of the specimen was suspended vertically with its lower end immersed in a reservoir of distilled water and the height of water reached in the fabric against gravity was visually observed and recorded after a fixed time. The initial and extended wicking rate, expressed in mm/s, indicates the average speed of water to reach 20 and 150 mm height, respectively.	- Initial wicking rate, 20 mm divided by time spent (mm/s)<br>- Extended wicking rate, 150 mm divided by time spent (mm/s)
Horizontal wicking test [4]	A fixed amount of water was supplied at the bottom side of fabric at a constant rate (10 ml/h). A camera, standing on top of the set up, was utilized to capture the image of the wetted sample and the water spreading area was measured.	- Horizontal wicking area (cm^2)
Moisture management tester (AATCC 195) [5]	The sample was put in-between two sets of metal electrodes and a fixed quantity of liquid was dropped onto the back side of the fabric and the direction of water spread was traced automatically by the metal electrodes.	- Overall (liquid) moisture management capability (OMMC)
Water absorption capacity test [4]	Fabric was put into a tank of water and 5 minutes was allowed for it to sink completely into the water. The fabric was then taken out by tweezers and hung onto a rod vertically until there was no water dripping within a 30-second interval. The water gain in fabric was measured and it is expressed as mass of water gain per unit gram of fabric in percentage.	- Wet pick-up (%)

Supplementary Table S2. Literatures showing the difference in sweat rate under different activities

Activity	Special dressing	Environmental condition			Sweat rate		Reference
		T_{db} (˚C)	R.H. %	Wind speed ($m \cdot s^{-1}$)	($ml \cdot h^{-1}$)	($ml \cdot m^{-2} \cdot h^{-1}$)	
Running at 10.9 $km \cdot h^{-1}$	-	30	33	2.4	800	491	Mora-Rodriguez et al. [6]
Running at 11.9 $km \cdot h^{-1}$	-	21.7	69	-	930	521	Kavanagh and shepherd [7]
Running at 13 $km \cdot h^{-1}$	-	30	33	2.4	1100	675	Mora-Rodriguez et al. [6]
Running at 13.9 $km \cdot h^{-1}$	-	28.5	72	-	1810	932	Millard-Stafford et al. [8]
Cycling at 70 % of VO_{2max}	-	3.6	70	0.7	550	293	Galloway and Maughan [9]
	-	10.5	70	0.7	650	346	
	-	20.6	70	0.7	780	415	
	-	30.5	70	0.7	1150	612	
Cycling (Hill climbing)	-	28.7	-	0.9	3530	1824	Bardis et al. [10]
Tennis	-	25.4	32	1.2	1510	751	Honrnery et al . [11]
	-	32.2	53.9	0.05-3.55	1740	902	Bergeron et al. [12]
Beach volleyball	-	33.6	56	-	1996	950	Zetou et al. [13]
Soccer	-	9.6	56.2	-	1027	521	Broad et al. [14]
	-	24.6	41.4	-	1209	613	Broad et al. [14]
	-	30	48	-	1680	925	Silva et al. [15]
Basketball	-	23.3	41.4	-	1601	704	Broad et al. [14]
Netball	-	22.1	66.1	-	982	512	Broad et al. [14]
American football	Half pads (shorts, shoulder pads, and helmets)	28.4	64.9	-	2147	888	Godek et al. [16]
	Full pads (adding hip, thigh, and knee pads)	28	-	-	2100	885	Godek et al. [17]
Ice-hockey	Standard dressing	12.2	44	-	1460	678	Palmer et al. [18]
Rally car driving	A FIA approved three-layer Nomex race suit and helmet, boots and gloves	50	-	-	1350	687	Walker et al. [19]
Walk continuously on a treadmill at 4.8 $km \cdot h^{-1}$, 2 % grade	Normal military combat clothing (4-5 kg, 1.4 clo, permeability index 0.44)	40	30	-	800	401	Aoyagi et al. [20]
	Nuclear, biological, and chemical hazards (NBC) protective clothing (8-9 kg, 2.4 clo, permeability index 0.33)	40	30	-	1260	631	
Walking on treadmill at 4.8 $km \cdot h^{-1}$, 4 % grade	Nuclear, biological and chemical (NBC) protective clothing (1.88 clo, permeability index 0.33)	40	30	<0.1	1540	797	McLellan and Cheung [21]

*Supplementary Table S3. Estimate of sweat rate under different level of work^*

Setting of syringe pump (ml/h)	Level of work	Examples of activity	Estimate of sweat rate	
			(ml/h)*	($ml/m^2/h$)
1	Very low	Sitting, standing	125	70
3	Low	Walking, driving	375	208
5	Moderate	Cycling	625	347
10	High	Running, ball games	1250	694
20	Very high	Cycling (hill climbing)	2500	1389
40	Extremely high	Maximal level of work of human being (last for short duration only)	5000	2778

^ With reference to Supplementary Table S2 online and various standards [22,23]

* Body surface area of human body is assumed to be 1.8 m^2 [24]

Specimen

24 types of fabrics, 12 X 12 cm, were conditioned in a standard atmosphere (20 ± 1 °C and 65±5 % RH) for at least 24 hours prior to testing.

Supplementary Table S4. Details and specifications of various fabrics

	Fabric details	Fabric code.	Construction		Fiber content	Fabric density		Weight (g/m²)	Thickness (mm)	Porosity
			Type	Structure		epi	ppi			
Group A	Plain cotton fabrics finished with different concentration of water repellent finish ⧖	0W	Woven	Plain	Cotton	140	75	121.95	0.288	0.7250
		0.5W				142	75	124.60	0.336	0.7592
		1W				141	75	124.00	0.340	0.7632
		2W ¶				140	74	124.95	0.340	0.7614
		3W ¶				142	75	124.70	0.344	0.7646
		5W ¶				142	74	124.40	0.356	0.7731
		10W ¶				142	75	124.90	0.360	0.7747
		20W ¶				141	74	124.50	0.356	0.7729
		60W ¶				140	74	125.20	0.352	0.7690
Group B	Thick shirting fabrics with different fabric structure and made by the same warp yarns but different weft yarns	2 §		2/2 twill		77	95	207.36	0.596	0.7741
		4 †				77	97	196.26	0.580	0.7803
		6 §		1/5 twill		78	89	220.52	0.828	0.8271
		8 †				80	89	201.08	0.800	0.8368
		12 §		2/2 rib		74	102	210.14	0.508	0.7314
		14 †				72	101	193.62	0.488	0.7424
		16 §		4/4 rib		69	106	204.08	0.724	0.8170
		18 †				68	106	191.34	0.712	0.8255
		20 §		Plain		73	73	179.28	0.472	0.7534
		22 †				76	74	163.54	0.432	0.7542
Group C	Fabrics with different structures and fibre compositions	PET		5/1 twill	Polyester	169	75	156.18	0.38	0.7043
		B		Satin	Viscose	191	121	123.60	0.260	0.6831
		D		Plain	Viscose	102	71	89.80	0.256	0.7661
		Ramie		Plain	Ramie	60	55	123.30	0.260	0.6838
		ElastiC		Plain	Cotton, Spandex, Polyester	134	88	213.10	0.568	0.7150

⧖ OLEOPHOBOL® CO was applied to the fabrics through pad-dry-cure process (The number in the fabric code indicates the concentration of finish in ml/L).

¶ Fabrics with water absorption time longer than 60 seconds measured by wettability test (AATCC 79) were defined as hydrophobic fabrics.

§ Fabrics made by 20/1 s cotton weft yarn (one coarser yarn)

† Fabrics made by 45/2 s cotton weft yarn (two thinner yarns twisted together)

Experimental setting - the selection of pressure loading

Effect of pressure loading on testing reproducibility

The reproducibility of the test was investigated by comparing the coefficient of variation (CV%) of three important parameters, namely fraction of water absorbed by bottom filter paper, transplanar ratio and wetted area of fabrics. From Supplementary Table S5, it indicated that the CV% of the three parameters decreases with the increase of pressure loading while the variation of the three parameters measured at 2.5 g/cm^2 and 4 g/cm^2 loading is lower than 5 % and does not have remarkable difference. It implies that 2.5 g/cm^2 and 4 g/cm^2 loading can give reproducible result.

Supplementary Table S5. Coefficient of variation of different measurements under different pressure loading

Pressure loading (g/cm^2) / Fabric Code.	Fraction of water absorbed in bottom filter paper			Transplanar ratio			Wetted area of fabrics		
	0.5	2.5	4	0.5	2.5	4	0.5	2.5	4
S1	9.37	3.44	1.70	8.50	3.55	3.50	2.32	3.76	7.49
S2	8.15	1.12	3.68	17.63	2.54	6.17	5.13	4.11	3.88
S3	6.19	3.81	2.22	13.30	8.63	5.18	6.61	3.70	4.20
S4	7.78	2.70	2.99	12.83	5.27	4.95	4.89	2.77	3.97
S5	10.43	4.16	2.18	17.49	8.10	1.87	29.19	13.27	7.17
S6	8.65	3.37	2.65	11.54	4.84	3.62	9.14	4.75	5.12
S7	6.43	2.29	3.73	8.56	4.28	3.09	8.81	4.49	4.49
S8	4.29	1.76	2.94	12.93	4.10	5.52	3.54	2.38	2.48
Average CV% of 8 fabrics	7.66	2.83	2.76	12.85	5.16	4.24	8.70	4.90	4.85

Effect of pressure loading on testing sensitivity

In order to investigate testing sensitivity, one-way between-subject ANOVA test was performed. This test aims to investigate if there are significant differences among the eight fabrics and the test results are shown in Supplementary Table S6. Actually, these are all 100% cotton fabrics with high absorption rate (i.e. water absorption time less than 3 sec as measured according to AATCC 79). Even though their hydrophilicity is similar, the FFWTT results show that there are significant differences in all cases ($p<0.05$), implying that FFWTT is sensitive irrespective of pressure loading. Supplementary Figure S1 further shows the degree of difference among the eight fabrics under different pressure loading. In general, the degree of difference reduces with pressure loading.

Supplementary Table S6. ANOVA test results among 8 fabrics at given loading pressure

**Pressure loading** (g/cm^2)	Fraction of water absorbed in bottom filter paper			Transplanar ratio			Wetted area of fabrics		
	0.5	2.5	4	0.5	2.5	4	0.5	2.5	4
p-value	.000	.000	.000	.000	.000	.000	.000	.000	.000

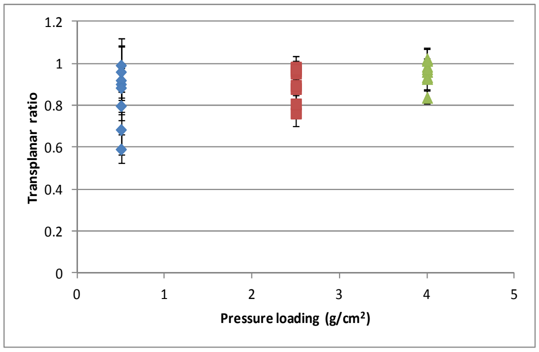

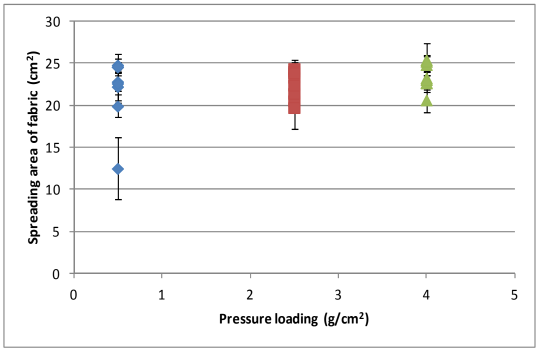

(a) **(b)**

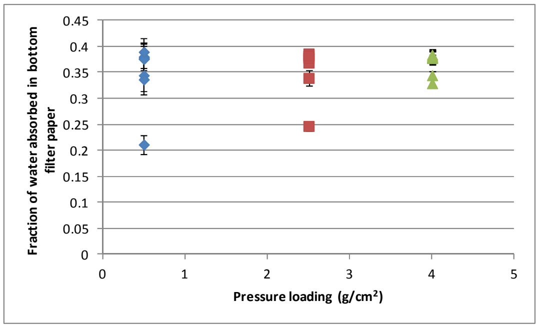

(c)

Supplementary Figure S1. Measurements under different pressure loading. (a) Transplanar ratio. (b) Spreading area of fabric, and (c) Fraction of water absorbed in bottom filter paper.

In general, a higher pressure loading may give highly reproducible results. However, a higher loading pressure may reduce the differences of testing results between different fabrics compared to a lower loading pressure.

Results and Discussion

Results for Group B's fabrics

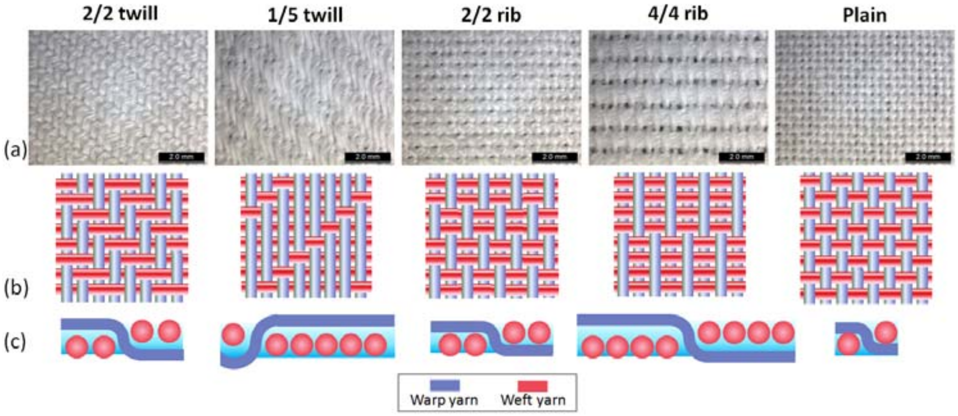

Supplementary Figure S2. Images of Group B's fabrics. (a): Microscopic images of fabrics with different weave pattern, including 2/2 twill (fabric '4'), 1/5 twill (fabric '8'), 2/2 rib (fabric '14'), 4/4 rib (fabric '18') and plain (fabric '22'). (b): Illustrate the weave pattern of fabrics from top view showing the arrangement of yarns. (c): Illustrate the cross-section of fabrics showing the arrangement of yarn.

Supplementary Table S7. Two-way ANOVA test result for Group B's fabric

		Fraction of water absorbed in bottom filter paper			Fraction of water absorbed in fabric			Transplanar ratio			Wetted area of fabric			Water content of fabric		
		3ml/h	10 ml/h	40 ml/h	3ml/h	10 ml/h	40 ml/h	3ml/h	10 ml/h	40 ml/h	3ml/h	10 ml/h	40 ml/h	3ml/h	10 ml/h	40 ml/h
Sig.	Fabric Structure	.000	.000	.000	.000	.000	.000	.056	.000	.000	.000	.000	.000	.000	.000	.000
	Yarn	.000	.000	.070	.000	.000	.272	.001	.000	.353	.000	.000	.000	.004	.000	.000
	Fabric Structure*Yarn	.000	.785	.496	.000	.033	.001	.000	.000	.000	.000	.464	.351	.240	.000	.002
R squared		.931	.848	.930	.946	.919	.962	.669	.788	.676	.884	.852	.697	.962	.943	.944

Supplementary Table S8. Results of Post hoc tests showing the mean difference of Group B's fabrics with different fabric structure

Multiple comparisons

(I)	(J)	Fraction of water absorbed in bottom filter paper			Fraction of water absorbed in fabric			Transplanar ratio			Wetted area of fabric (cm^2)			Water content of fabric		
		3ml/h	10 ml/h	40 ml/h	3ml/h	10 ml/h	40 ml/h	3ml/h	10 ml/h	40 ml/h	3ml/h	10 ml/h	40 ml/h	3ml/h	10 ml/h	40 ml/h
2/2 twill	1/5 twill	0.066*	0.039*	0.057*	-0.123*	-0.104*	-0.105*	-0.001	0.116*	-0.013	-5.29*	-3.07*	-1.96	0.065*	0.073*	0.072*
	2/2 rib	-0.006	-0.050*	-0.019*	0.025	0.109*	0.054*	-0.042	-0.054	-0.064	0.26	4.37*	0.14	-0.033*	-0.052*	-0.038*
	4/4 rib	0.062*	0.063*	0.040*	-0.100*	-0.096*	-0.078*	-0.060	-0.055	0.010	-4.54*	-4.56*	-2.37*	0.047*	0.059*	0.058*
	plain	-0.046*	-0.063*	-0.030*	0.092*	0.138*	0.085*	-0.027	-0.065	-0.102*	1.66	2.10	-2.23	-0.016*	-0.007	-0.004
1/5 twill	2/2 twill	-0.066*	-0.039*	-0.057*	0.123*	0.104*	0.105*	0.001	-0.116*	0.013	5.29*	3.07*	1.96	-0.065*	-0.073*	-0.072*
	2/2 rib	-0.072*	-0.089*	-0.076*	0.148*	0.213*	0.159*	-0.041	-0.170*	-0.051	5.55*	7.44*	2.09	-0.098*	-0.125*	-0.109*
	4/4 rib	-0.004	0.025	-0.017*	0.023	0.008	0.027*	-0.060	-0.170*	0.023	0.75	-1.49	-0.42	-0.019*	-0.013	-0.014
	plain	-0.112*	-0.101*	-0.087*	0.215*	0.241*	0.190*	-0.026	-0.181*	-0.089*	6.95*	5.17*	-0.27	-0.081*	-0.080*	-0.076*
2/2 rib	2/2 twill	0.006	0.050*	0.019*	-0.025	-0.109*	-0.054*	0.042	0.054	0.064	-0.26	-4.37*	-0.14	0.033*	0.052*	0.038*
	1/5 twill	0.072*	0.089*	0.076*	-0.148*	-0.213*	-0.159*	0.041	0.170*	0.051	-5.55*	-7.44*	-2.09	0.098*	0.125*	0.109*
	4/4 rib	0.068*	0.113*	0.059*	-0.124*	-0.206*	-0.132*	-0.019	0.000	0.074	-4.80*	-8.92*	-2.51*	0.080*	0.111*	0.095*
	plain	-0.040*	-0.013	-0.011	0.067*	0.028	0.031*	0.015	-0.010	-0.038	1.40	-2.26	-2.37*	0.017*	0.045*	0.034*
4/4 rib	2/2 twill	-0.062*	-0.063*	-0.040*	0.100*	0.096*	0.078*	0.060	0.055	-0.010	4.54*	4.56*	2.37*	-0.047*	-0.059*	-0.058*
	1/5 twill	0.004	-0.025	0.017*	-0.023	-0.008	-0.027*	0.060	0.170*	-0.023	-0.75	1.49	0.42	0.019*	0.013	0.014
	2/2 rib	-0.068*	-0.113*	-0.059*	0.124*	0.206*	0.132*	0.019	0.000	-0.074	4.80*	8.92*	2.51*	-0.080*	-0.111*	-0.095*
	plain	-0.108*	-0.126*	-0.070*	0.192*	0.234*	0.163*	0.034	-0.010	-0.111*	6.20*	6.66*	0.14	-0.063*	-0.066*	-0.062*
plain	2/2 twill	0.046*	0.063*	0.030*	-0.092*	-0.138*	-0.085*	0.027	0.065	0.102*	-1.66	-2.10	2.23	0.016*	0.007	0.004
	1/5 twill	0.112*	0.101*	0.087*	-0.215*	-0.241*	-0.190*	0.026	0.181*	0.089*	-6.95*	-5.17*	0.27	0.081*	0.080*	0.076*
	2/2 rib	0.040*	0.013	0.011	-0.067*	-0.028	-0.031*	-0.015	0.010	0.038	-1.40	2.26	2.37*	-0.017*	-0.045*	-0.034*
	4/4 rib	0.108*	0.126*	0.070*	-0.192*	-0.234*	-0.163*	-0.034	0.010	0.111*	-6.20*	-6.66*	-0.14	0.063*	0.066*	0.062*

* The mean difference is significant at $\alpha=0.05$ level with Scheffé's method applied.

Accuracy of FFWTT

In order to check the accuracy of the proposed set up, the FFWTT results was correlated with and the newly developed Spontaneous Uptake Water Transport Tester (SUWTT) [1] and other conventional measurement methods, including wettability test (AATCC 79) [2], vertical wicking test (AATCC 197) [3], horizontal wicking test [4], moisture management test (MMT) (AATCC 195) [5] and water absorption capacity test [4]. For better and easy understanding about these methods, their testing principles are summarized in Supplementary Table S1. The results of the correlation analysis are demonstrated in Supplementary Figure S3.

The correlation between FFWTT and SUWTT is shown in Supplementary Figure S3(a) to S2(e). For both of the tests, the test sample was put in-between two filter papers and water distribution in each layer, wetted area and water content of fabric, and transplanar ratio were measured. Supplementary Figure S3(a) shows that the mass of water absorbed by fabric in SUWTT has strong and positive correlation with the fraction of water absorbed by fabric in FFWTT. Additionally, the mass of water absorbed by bottom filter paper in SUWTT has strong and positive correlation with the fraction of water absorbed by bottom filter paper in FFWTT, as illustrated in Supplementary Figure S3(b). Supplementary Figure S3(c) shows that the wetted area of fabric by SUWTT has strong and positive correlation with the wetted area of fabric by FFWTT measured at 10 ml/h water flow (adj. R^2=0.80) and 40 ml/h water flow (adj. R^2=0.88), but only moderate correlation with the FFWTT result measured at 3 ml/h water supply (adj. R^2=0.61). Similarly, as shown in Supplementary Figure S3(d), the water content of fabric by SUWTT is strongly correlated with the water content of fabric by FFWTT measured at 40 ml/h water supply (adj. R^2=0.94), but is only moderately correlated with the FFWTT result measured at 3 ml/h water flow (adj. R^2=0.60) and 10 ml/h water supply (adj. R^2=0.66). Since water absorption and transport property of fabrics is sweat-rate dependent, this explain the difference in correlation coefficient value observed under different water flow rate. For the 3-layer SUWTT test, the rate of water supply, ranging from 37.37 g/h to 80.66 g/h as shown in Supplementary Figure S5 online, varies with water absorption capacity of fabrics and is rather high. Hence, SUWTT has higher correlation with FFWTT under higher water flow rate (i.e. 40 ml/h water supply). On the other hand, transplanar ratio by SUWTT has strong correlation with transplanar ratio by FFWTT as illustrated in Supplementary Figure S3(e).

Fraction of water absorbed by fabric in the FFWTT test has strong and positive correlation with water absorption capacity of fabric (as shown in Supplementary Figure S3(f)) and initial wicking rate (as shown in Supplementary Figure S3(g)). Fabrics with higher water absorption capacity or faster absorption contribute to higher amount of water absorbed by fabric. Figure S3(h) shows a clear and positive correlation between transplanar ratio by FFWTT and overall moisture management capability (OMMC) by MMT. All in all, these figures prove that FFWTT can give accurate result.

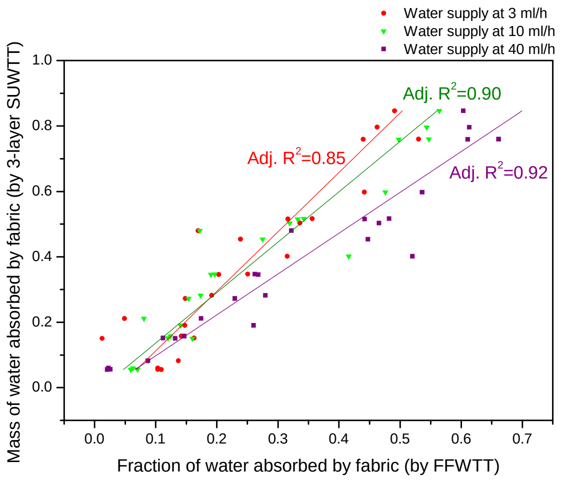
Water supply at 3 ml/h
Water supply at 10 ml/h
Water supply at 40 ml/h
Adj. R²=0.90
Adj. R²=0.85
Adj. R²=0.92
Mass of water absorbed by fabric (by 3-layer SUWTT)
Fraction of water absorbed by fabric (by FFWTT)

(a)

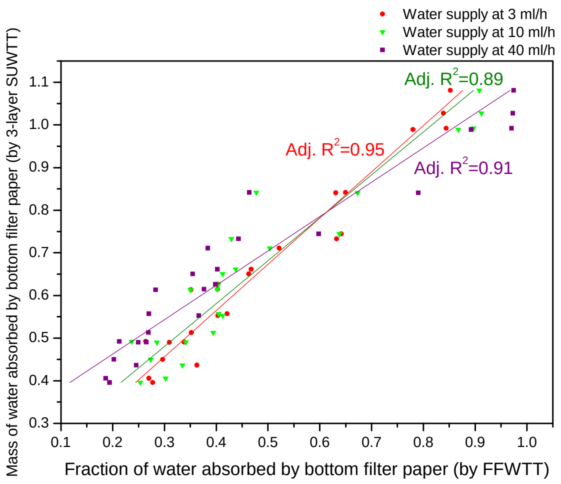
Water supply at 3 ml/h
Water supply at 10 ml/h
Water supply at 40 ml/h
Adj. R²=0.89
Adj. R²=0.95
Adj. R²=0.91
Mass of water absorbed by bottom filter paper (by 3-layer SUWTT)
Fraction of water absorbed by bottom filter paper (by FFWTT)

(b)

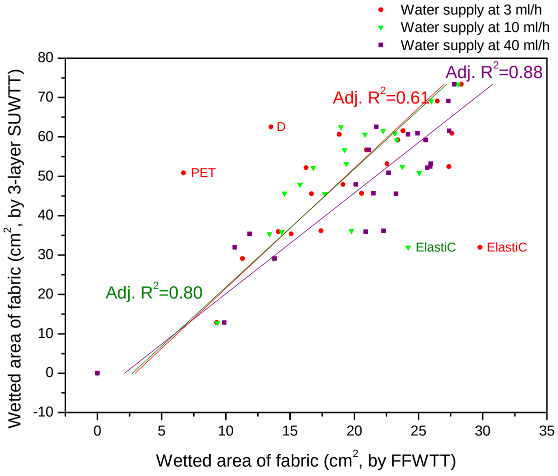
Water supply at 3 ml/h
Water supply at 10 ml/h
Water supply at 40 ml/h
Adj. R²=0.88
Adj. R²=0.61
D
PET
ElasticC
ElasticC
Adj. R²=0.80
Wetted area of fabric (cm², by 3-layer SUWTT)
Wetted area of fabric (cm², by FFWTT)

(c)

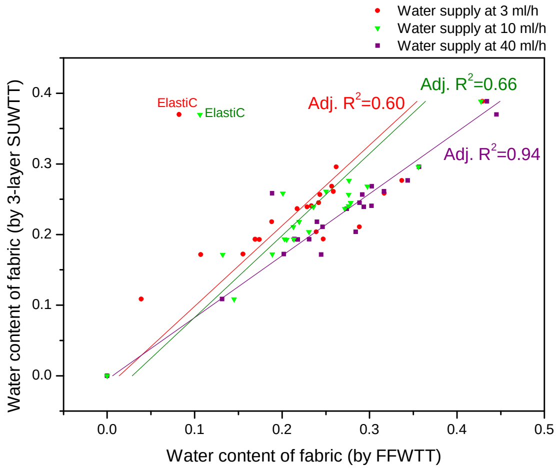
Water supply at 3 ml/h
Water supply at 10 ml/h
Water supply at 40 ml/h
Adj. R²=0.66
ElasticC
ElasticC
Adj. R²=0.60
Adj. R²=0.94
Water content of fabric (by 3-layer SUWTT)
Water content of fabric (by FFWTT)

(d)

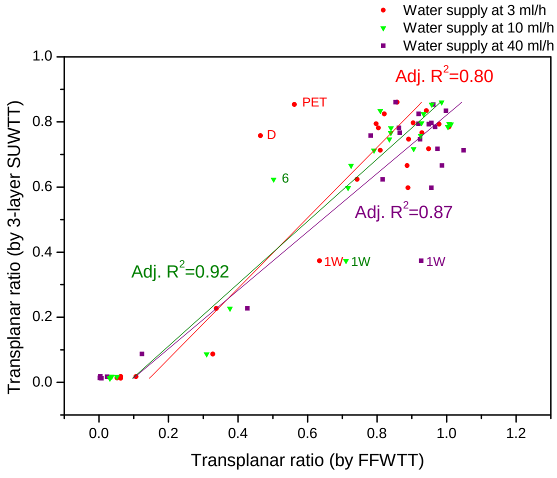
Water supply at 3 ml/h
Water supply at 10 ml/h
Water supply at 40 ml/h
Adj. R²=0.80
PET
D
6
Adj. R²=0.87
Adj. R²=0.92
1W
1W
1W
Transplanar ratio (by 3-layer SUWTT)
Transplanar ratio (by FFWTT)

(e)

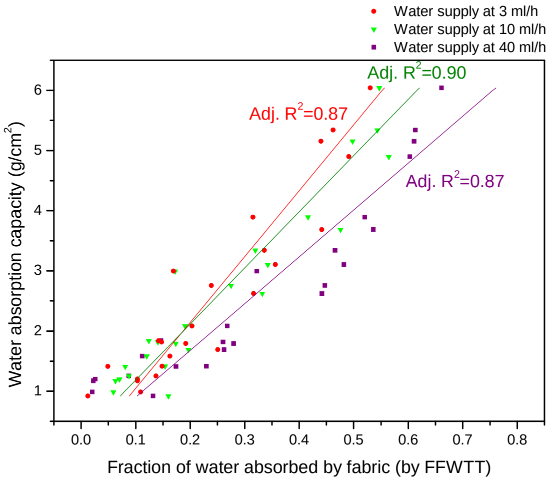
Water supply at 3 ml/h
Water supply at 10 ml/h
Water supply at 40 ml/h
Adj. R²=0.90
Adj. R²=0.87
Adj. R²=0.87
Water absorption capacity (g/cm²)
Fraction of water absorbed by fabric (by FFWTT)

(f)

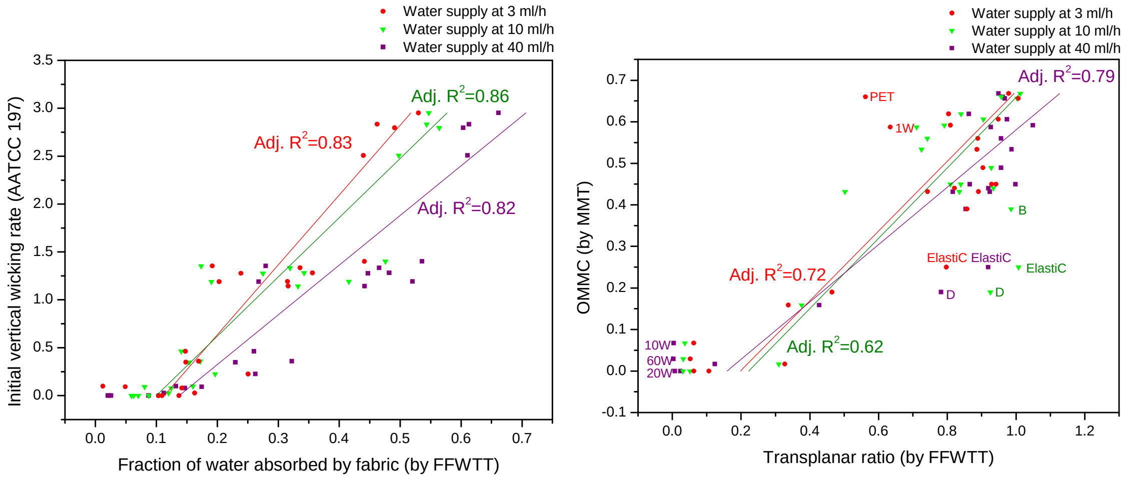

(g) (h)

Supplementary Figure S3. Scatter plot showing the correlation between FFWTT test and various conventional water absorption measurements. The red circles show the data measured at 3 ml/h water flow while green triangles and purple squares denote the result measured at 10 ml/h and 40 ml/h water flow, respectively. These figures were fitted with linear function.

(a): Mass of water absorbed by fabric in SUWTT against fraction of water absorbed by fabric in FFWTT. (b): Mass of water absorbed by bottom filter paper in SUWTT against fraction of water absorbed by bottom filter paper in FFWTT. (c): Wetted area of fabric by SUWTT against wetted area of fabric by FFWTT. (d): Water content of fabric by SUWTT against water content of fabric by FFWTT. (e): Transplanar ratio by SUWTT against transplanar ratio by FFWTT. (f): Water absorption capacity against fraction of water absorbed by fabric by FFWTT. (g) Initial vertical wicking rate against fraction of water absorbed by fabric by FFWTT. (h): OMMC by MMT against transplanar ratio by FFWTT.

Validity of FFWTT

In order to examine the validity of the instrument, the FFWTT results were correlated with the subjective wetness sensation and it was assessed in accordance with Tang et al.'s method [25]. In their study, water was applied to the fabric at a constant flow rate and the amount of water required to trigger wet sensation, which depends on wetting and wicking property of the fabric, was evaluated. The much the water required to trigger wet sensation, the more comfortable the fabric is. They found that human is not capable of differentiating between hydrophilic and hydrophobic fabrics, and either one (i.e. hydrophilic or hydrophobic groups) should be interpreted at a time. Wettability test (AATCC 79) was performed to define the hydrophobicity of these fabrics. Fabrics whose water absorption time exceeds 60 seconds are defined as hydrophobic while hydrophilic fabric refers to absorbing substrate with water absorption time shorter than 60 seconds. As a result, these 24 fabrics were separated into two groups and hydrophobic fabrics were eliminated for further analysed. Supplementary Figure S4(a) shows that fraction of water absorbed by hydrophilic fabric and absolute threshold amount of water required to trigger wet sensation is

strongly and positively correlated, suggesting that FFWTT has high validity in estimating subjective wetness sensation.

Apart from the psychophysical measurement, the percentage of water left on skin after the subjective test (as calculated by equation (1)), an indirect physical measurement reflecting skin wetness, was correlated with FFWTT results. Supplementary Figure S4(b) shows that the percentage of water left on skin has positive relationship with the fraction of water absorbed by bottom filter paper by FFWTT. The direction of correlation is rational and it implies that the bottom filter paper can simulate our skin condition quite well.

$$\text{Percentage of residual water left on skin, } P_W\ (\%) = \frac{\text{Injection amount (g)} - \text{Amount of water absorbed by fabric (g)}}{\text{Injection amount (g)}} \times 100\% \qquad (1)$$

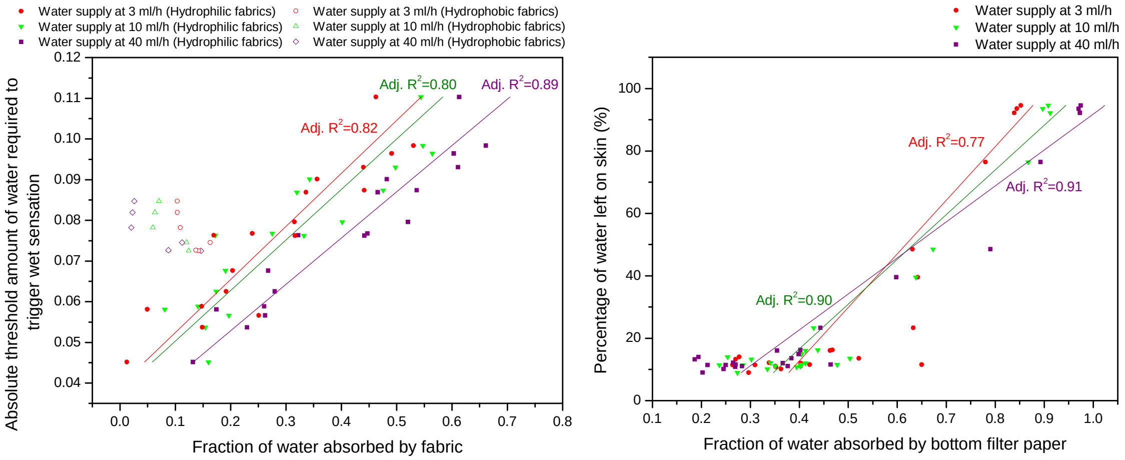

(a) (b)

Supplementary Figure S4. Scatter plot showing the correlation between FFWTT test and subjective assessment on wetness sensation. The red circles show the data measured at 3 ml/h water flow while green triangles and purple squares denote the result measured at 10 ml/h and 40 ml/h water flow, respectively. (a): Absolute threshold amount of water required to trigger wet sensation was plotted against fraction of water absorbed by fabric layer in FFWTT. The data points were fitted with linear function while the hydrophobic fabrics (marked in hollow shape) were eliminated from the model. The reason for not including these samples into the model is that people cannot differentiate the wetness level between hydrophilic and hydrophobic samples. This phenomenon can attribute to the different mechanisms involved to evoke the wet sensation. For hydrophilic fabrics, water spreads widely, evaporates quickly (dissipates body heat) and sticks closely to the skin which stimulates the cold receptors and mechanoreceptors and so boosting the wet sensation. For hydrophobic fabrics, wet sensation may attribute to the formation of water film between fabric-skin interface or the detection of dipping water. (b): Percentage of water left on skin after subjective assessment was plotted against fraction of water absorbed by bottom filter paper in FFWTT. The data points were fitted with linear function. The amount of water left on skin depends on water absorption and transport properties of the fabrics. For those hydrophobic fabrics, water cannot be absorbed by the fabric and majority of water may leave on skin, contributing to higher percentage of water left on skin. Inversely, for the hydrophilic fabrics, water was readily absorbed by the fabric contributing to lower percentage of water left on skin.

Uncertainty of different measurement parameters

Some parameters such as water content and transplanar ratio cannot be measured in a single measurement. In fact, several physical factors are involved and the uncertainty of these parameters can be calculated from the uncertainty of each direct measurement [26]. For the fraction of water absorbed by each layer, the water absorption amount by a specific layer is divided by and interrelated with the total absorption amount of the three layers, so the uncertainty of a specific layer is calculated by the sum of fractional uncertainties in two direct measurements, as shown in equation (2). For the transplanar ratio, the water absorption amount by top filter paper is divided by and interrelates with the water absorption amount in the bottom filter paper, so the uncertainty of transplanar ratio is calculated similarly by equation (3). The calculation of uncertainty of water content is based on the assumption that all errors are independent and random, and it is computed by the quadratic sum according to equation (4). The average uncertainty of each parameter is summarised in Supplementary Table S9. When considering the hydrophobic fabrics, the uncertainty of these parameters is particularly high. It might be due to the little amount of water absorbed by the fabric as well as by the top filter paper.

$$\text{Uncertainty of fraction of water absorbed by a specific layer} \tag{2}$$
$$= \frac{\delta_{\text{Mass of water absorbed by a specific layer}}}{\text{Mass of water absorbed by a specific layer}} + \frac{\delta_{\text{Total absorption mass by the three layers}}}{\text{Total absorption mass by the three layers}}$$

$$\text{Uncertainty of Transplanar ratio} \tag{3}$$
$$= \frac{\delta_{\text{Fraction of water absorbed by Filter paper}_{\text{Top}}}}{\text{Fraction of water absorbed by Filter paper}_{\text{Top}}} + \frac{\delta_{\text{Fraction of water absorbed by Filter paper}_{\text{Bottom}}}}{\text{Fraction of water absorbed by Filter paper}_{\text{Bottom}}}$$

$$\text{Uncertainty of Water content} \tag{4}$$
$$= \sqrt{\left(\frac{\delta_{\text{Mass of water absorbed}}}{\text{Mass of water absorbed}}\right)^2 + \left(\frac{\delta_{\text{Wetted area}}}{\text{Wetted area}}\right)^2 + \left(\frac{\delta_{\text{Thickness}}}{\text{Thickness}}\right)^2 + \left(\frac{\delta_{\text{Porosity}}}{\text{Porosity}}\right)^2}$$

where δ is the measured uncertainty from each component.

Supplementary Table S9. Uncertainty (%) of different parameters

	Water flow rate								
	3ml/h			10 ml/h			40 ml/h		
	Hydrophilic fabrics	Hydrophobic fabrics ^^	24 types of fabrics	Hydrophilic fabrics	Hydrophobic fabrics ^^	24 types of fabrics	Hydrophilic fabrics	Hydrophobic fabrics ^^	24 types of fabrics
Fraction of water absorbed by fabric	7.7	7.0	7.5	8.9	9.6	9.1	6.2	11.3	7.5
Fraction of water absorbed by bottom filter paper	4.9	4.8	4.9	6.8	4.8	6.3	6.3	3.3	5.5
Transplanar ratio	8.0	9.7	8.4	10.0	19.6	12.4	9.5	39.4	17.0
Water content of fabric	9.7	10.7	9.7	11.2	11.5	11.3	7.6	16.0	9.2

^^ Including fabric '2W', '3W', '5W', '10W', '20W' and '60W'.

Reproducibility of the test

To ensure the reproducibility of the test result, it is necessary to handle the fabric carefully and in a repeatable manner. Different parts of the instrument such as the tube, plastic plate and syringe pump should be calibrated properly. The tube diameter, tube length, the size of the hole in the plastic plate, the height difference between the syringe pump and the plastic plate from level may contribute to the variability of the result and a constant setting of the set up should be maintained. In order to check the reproducibility of the instrument, this may be done by supplying water at a constant speed through the syringe pump, collecting the effluent by a standard material for a pre-determined time, and weighing. For the standard material, the collected amount of water is within 3% coefficient of variation (CV) of the pre-determined amount, we could conclude that the setting is well-calibrated.

The CV % of various measured parameters by the FFWTT is summarized in Supplementary Table S10. Except for transplanar ratio, the CV % of the remaining parameters is relatively low. Regarding the transplanar ratio, the average CV % for the 24 fabrics is 6.08 % for water flow rate of 3 ml/h, 9.64 % for 10 ml/h and 14.69 % for 40 ml/h, which is comparatively high. For the hydrophobic fabrics, there is only little water transport to the top filter paper and a slight deviation in it would cause a large variation in transplanar ratio. Hence, the average CV % for the transplanar ratio of the hydrophobic fabrics is 8.37 % for water flow rate of 3 ml/h, 18.58 % for 10 ml/h and 39.03 % for 40 ml/h.

These findings support that FFWTT is more favourable for testing hydrophilic medium in terms of reproducibility. But still, when testing the hydrophobic fabrics, the CV % is acceptable for the fraction of water absorbed by the bottom filter paper, fraction of water absorbed by fabric and wetted area of fabric, implying that this method is suitable for the hydrophobic medium as well.

Supplementary Table S10. Coefficients of variation (%) of FFWTT result

	Water flow rate								
	3ml/h			10 ml/h			40 ml/h		
	Hydrophilic fabrics	Hydrophobic fabrics ^	24 types of fabrics	Hydrophilic fabrics	Hydrophobic fabrics ^	24 types of fabrics	Hydrophilic fabrics	Hydrophobic fabrics ^	24 types of fabrics
Fraction of water absorbed by fabric	5.56	5.05	5.43	6.58	7.76	6.88	4.49	11.63	6.28
Fraction of water absorbed by bottom filter paper	3.53	1.46	3.01	4.93	2.25	4.26	4.23	1.50	3.55
Transplanar ratio	5.32	8.37	6.08	6.66	18.58	9.64	6.57	39.03	14.69
Wetted area of fabric	6.83	8.64	7.09	8.05	7.40	7.96	5.18	11.83	6.13
Water content of fabric	5.11	6.04	5.24	4.90	9.71	5.59	4.39	9.05	5.05

^ Including Fabric '2W', '3W', '5W', '10W', '20W' and '60W'

Comparisons of FFWTT against SUWTT

Supplementary Table S11. Comparisons of FFWTT against SUWTT

	FFWTT	SUWTT
Principle of the instrument	Gravimetric and image analysis technique	
Principle of water supply	Forced flow of water supply	Demand wetting
Rate of water supply	Sweat rate-dependent	Fabric-dependent (as shown in Supplementary Figure S5)
Measurement parameters	- Fraction of water absorbed by fabric<br>- Fraction of water absorbed by bottom filter paper<br>- Wetted area of fabric<br>- Water content of fabric<br>- Transplanar ratio	1-layer test<br>- Water absorption rate of fabric<br>- Wetted area of fabric<br>- Water content of fabric<br>3-layer test<br>- Mass of water absorbed by fabric<br>- Mass of water absorbed by bottom filter paper<br>- Transplanar ratio
Specific equipment/component required	Syringe pump<br>Balance<br>Image analysis component	Motor<br>Photoelectric sensor<br>Water pump<br>Water tank<br>Data acquisition (DAQ) system<br>Balance<br>Image analysis component
Sample size	12 X 12 cm	

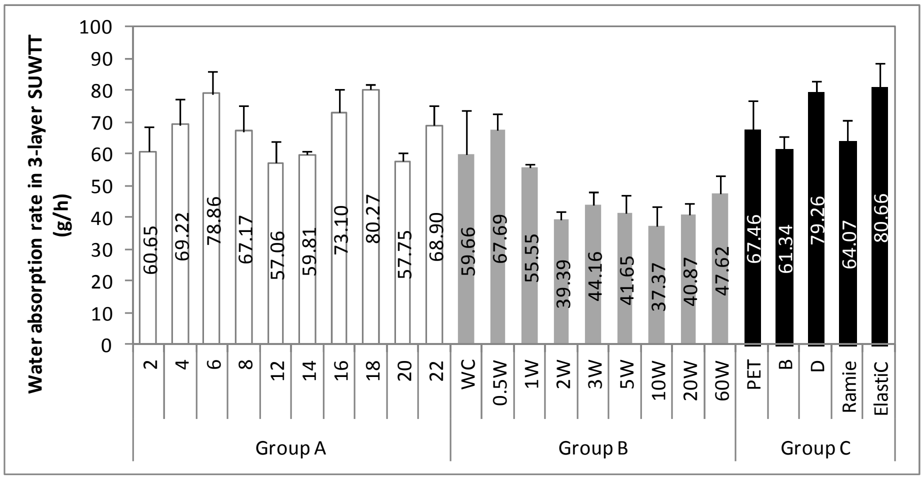

Supplementary Figure S5. Water absorption rate of fabrics by 3-layer SUWTT test. The bars in white colour indicate the result for group A's samples while the bars in grey and black denotes group B's and group C's samples, respectively. The error bars represent mean ± S.D. of five samples. From the figure, it can be observed that the water absorption rate of fabrics is fabric-dependent and this is totally different from FFWTT.

Summary

Supplementary Table S12. Comparisons with the conventional testing methods

Test method	Limitations	Problem solved by the proposed method?
Spontaneous Uptake Water Transport Tester (SUWTT)	The rate of water supply does not simulate the actual wear condition	✓
Drop test (AATCC 79)	Does not simulate the profuse sweating condition	✓
	The determination of end-point of testing is prone to subjective variation	✓
Vertical wicking test (AATCC 197)	The direction of water transport does not simulate actual use	✓
Vertical wicking test (AATCC 197); Horizontal wicking test (AATCC 198)	The difficulty in tracing the position of liquid front by observation	Optical scanner was used to capture the digital image of the wetted pattern. The detection of liquid front is done by computer. As same as AATCC 197 and AATCC 198, it may be difficult for some particular fabrics (e.g. dark-coloured fabric), but computer deems to have better judgment than human eyes and the calculation of wetted area will be more precise.
Horizontal wicking test (AATCC 198); Moisture Management Tester (AATCC 195)	Water was applied from the top of fabric and gravity induced pressure may affect the wicking property	✓
Moisture Management Tester (AATCC 195)	Not suitable for fabrics with rough surface and thin fabrics and sensitivity may not be good enough for thin fabric	✓
	Not suitable for hydrophobic fabrics	✓
Contact angle measurement	Not applicable to material which absorb water very fast	✓
Gravimetric Absorption Testing System (GATS); Transplanar Water Transport Tester (TWTT)	Cannot differentiate the direction of water flow within the fabric	✓
Transplanar Water Transport Tester (TWTT)	A slight deviation on the evenness of surface might affect the contact with the plate, thus affecting the accuracy of result	✓
	Only one layer of fabric could be tested	✓
Water absorption capacity measurement	Fabric saturation is considered, but the initial wetting performance is not examined.	The initial water absorption rate cannot be measured.
Spectroscopic method (e.g. MRI and NMR)	Expensive equipment and complicated handling	✓

References

1 K. P. M. Tang, Y.S. Wu, K. H. Chau, C. W. Kan & J. T. Fan. Characterizing the transplanar and in-plane water transport property of textiles with gravimetric and image analysis technique: Spontaneous Uptake Water Transport Tester. *Sci. Rep.* **5**, 9689, (2015).

2 *AATCC 79 Absorbency of textiles*. (American Association of Textile Chemists and Colorists, 2007).

3 *AATCC 197 Vertical wicking of Textiles*. (American Association of Textile Chemists and Colorists, 2011).

4 Tang, K. P. M., Kan, C. W. & Fan, J. T. Assessing and Predicting the Subjective Wetness Sensation of Textiles: Subjective and Objective Evaluation. *Text. Res. J.* **85**, 838-849, (2014).

5 *AATCC 195 Liquid Moisture Management Properties of Textile fabrics*. (American Association of Textile Chemists and Colorists, 2009).

6 Mora-Rodriguez, R., Ortega, J. & Hamouti, N. In a hot–dry environment racewalking increases the risk of hyperthermia in comparison to when running at a similar velocity. *Eur. J. Appl. Physiol.* **111**, 1073-1080, (2011).
7 Kavanagh, T. & Shephard, R. On the choice of fluid for the hydration of middle-aged marathon runners. *Med. Sci. Sports Exerc.* **11**, 26-35, (1977).
8 Millard-Stafford, M. L., Sparling, P. B., Rosskopf, L. B. & DiCarlo, L. J. Carbohydrate-electrolyte replacement improves distance running performance in the heat. *Med. Sci. Sports Exerc.* **24**, 934-940, (1992).
9 Galloway, S. & Maughan, R. J. Effects of ambient temperature on the capacity to perform prolonged cycle exercise in man. *Med. Sci. Sports Exerc.* **29**, 1240-1249, (1997).
10 Bardis, C. N., Kavouras, S. A., Arnaoutis, G., Panagiotakos, D. B. & Sidossis, L. S. Mild Dehydration and Cycling Performance During 5-Kilometer Hill Climbing. *J. Athl. Train.* **48**, 741-747, (2013).
11 Hornery, D. J., Farrow, D. & Mujika, I. An integrated physiological and performance profile of professional tennis. *Med. Sci. Sports Exerc.* **41**, 531-536, (2007).
12 Bergeron, M. F. *et al.* Fluid-electrolyte balance associated with tennis match play in a hot environment. *Int. J. Sport Nutr.* **5**, 180-180, (1995).
13 Zetou, E., Giatsis, G., Mountaki, F. & Komninakidou, A. Body weight changes and voluntary fluid intakes of beach volleyball players during an official tournament. *J. Sci. Med. Sport* **11**, 139-145.
14 Broad, E. M., Burke, L. M., Cox, G. R., Heeley, P. & Riley, M. Body weight changes and voluntary fluid intakes during training and competition sessions in team sports. *Int. J. Sport Nutr.* **6**, 307-320, (1996).
15 Da Silva, R. P. *et al.* Pre-game hydration status, sweat loss, and fluid intake in elite Brazilian young male soccer players during competition. *J. Sports Sci.* **30**, 37-42, (2012).
16 Godek, S. F., Bartolozzi, A. R. & Godek, J. J. Sweat rate and fluid turnover in American football players compared with runners in a hot and humid environment. *Med. Sci. Sports Exerc.* **39**, 205-211, (2005).
17 Godek, S. F. *et al.* Fluid Consumption and Sweating in National Football League and Collegiate Football Players With Different Access to Fluids During Practice. *J. Athl. Train.* **45**, 128-135, (2010).
18 Palmer, M. S., Logan, H. M. & Spriet, L. L. On-ice sweat rate, voluntary fluid intake, and sodium balance during practice in male junior ice hockey players drinking water or a carbohydrate-electrolyte solution. *Appl. Physiol. Nutr. Metab.* **35**, 328-335, (2010).
19 Walker, S. M., Dawson, B. & Ackland, T. R. Performance enhancement in rally car drivers via heat acclimation and race simulation. *Comp. Biochem. Physiol. A. Mol. Integr. Physiol.* **128**, 701-707, (2001).
20 Aoyagi, Y., McLellan, T. M. & Shephard, R. J. Effects of endurance training and heat acclimation on psychological strain in exercising men wearing protective clothing. *Ergonomics* **41**, 328-357, (1998).
21 McLellan, T. M. & Cheung, S. S. Impact of fluid replacement on heat storage while wearing protective clothing. *Ergonomics* **43**, 2020-2030, (2000).
22 *ISO 8996 Ergonomics of the thermal environment - Determination of metabolic rate*. (International Organization for Standardization, 2004).
23 *ISO 7933 Ergonomics of the thermal environment - Analytical determination and interpretation of heat stress using calculation of the predicted heat strain*. (International Organization for Standardization, 2004).
24 Sauer, H. J. in *Principles of heating, ventilating, and air conditioning : a textbook with design data based on the 2001 ASHRAE handbook--Fundamentals* (ed Jr. Harry J. Sauer, Ronald H. Howell, William J. Coad.) Ch. 8, 8.1-8.29 (American Society of Heating, Refrigerating and Air-Conditioning Engineers, 2001).
25 K. P. M. Tang, C. W. Kan & J. T. Fan. Psychophysical measurement of wet and clingy sensation of fabrics by the novel forearm test. *J. Sens. Stud.*, Under review, (2015).
26 Taylor, J. R. [An Introduction to Error Analysis : The Study of Uncertainties in Physical Measurements] [John R. Taylor (ed.)] [45-92] (University Science Books, United States, 1997).
